# Prediction of bacterial type IV secreted effectors by C-terminal features

**DOI:** 10.1186/1471-2164-15-50

**Published:** 2014-01-21

**Authors:** Yejun Wang, Xiaowei Wei, Hongxia Bao, Shu-Lin Liu

**Affiliations:** 1 Genomics Research Center, Harbin Medical University, Harbin, China; 2 Department of Microbiology and Infectious Diseases, University of Calgary, Calgary, Canada

**Keywords:** Type IV secretion system, Effector, Secretion signal prediction, Sequence analysis, Machine learning, *Helicobacter pylori*

## Abstract

**Background:**

Many bacteria can deliver pathogenic proteins (effectors) through type IV secretion systems (T4SSs) to eukaryotic cytoplasm, causing host diseases. The inherent property, such as sequence diversity and global scattering throughout the whole genome, makes it a big challenge to effectively identify the full set of T4SS effectors. Therefore, an effective inter-species T4SS effector prediction tool is urgently needed to help discover new effectors in a variety of bacterial species, especially those with few known effectors, e.g., *Helicobacter pylori*.

**Results:**

In this research, we first manually annotated a full list of validated T4SS effectors from different bacteria and then carefully compared their C-terminal sequential and position-specific amino acid compositions, possible motifs and structural features. Based on the observed features, we set up several models to automatically recognize T4SS effectors. Three of the models performed strikingly better than the others and T4SEpre_Joint had the best performance, which could distinguish the T4SS effectors from non-effectors with a 5-fold cross-validation sensitivity of 89% at a specificity of 97%, based on the training datasets. An inter-species cross prediction showed that T4SEpre_Joint could recall most known effectors from a variety of species. The inter-species prediction tool package, T4SEpre, was further used to predict new T4SS effectors from *H. pylori*, an important human pathogen associated with gastritis, ulcer and cancer. In total, 24 new highly possible *H. pylori* T4S effector genes were computationally identified.

**Conclusions:**

We conclude that T4SEpre, as an effective inter-species T4SS effector prediction software package, will help find new pathogenic T4SS effectors efficiently in a variety of pathogenic bacteria.

## Background

Type IV secretion system (T4SS) is a membrane-associated multi-component transporter complex, which plays important roles both in horizontal DNA transfer between different bacteria and in bacterial pathogenesis by translocating pathogenic substrates (DNA or protein) into host plant, animal or human cells [1,2]. A large number of T4SSs have been identified in a variety of bacterial species [1,2]. In many cases T4SSs have been implicated in protein delivery during the infection process, such as *Helicobacter* Cag-T4SS in human gastric ulcer and cancer, *Legionella* Dot/lcm-T4SS in Legionnaires’ disease, and *Bartonella* VirB/VirD4 in cat scratch disease [3-8]. Recently, a large-scale screening study performed in over 1000 prokaryotic genomes disclosed a total of 949 T4SSs, among which 267 lacked relaxases and were considered as putative protein-exporting T4SSs, since the T4SSs involving horizontal DNA transfer require the activity of relaxases while known protein-exporting T4SSs do not need relaxes [9].

The proteins specifically secreted through T4SS conduit are called type IV secreted (T4S) effectors, which exert important functions in cytoplasm of infected eukaryotic cells [10,11]. A large number of T4S effectors have been characterized experimentally with assays involving genetic complementation, reporter protein fusion, secretion apparatus or chaperone interaction, etc. [12-16]. However, it is difficult and time-consuming to find new effectors purely based on experimental methods. Additionally, in a given bacterial strain, most effectors are scattered throughout the genome rather than clustered in a narrow genomic region. Moreover, the validated effectors in different species are significantly diverse in sequence. Therefore, the bioinformatic methods so far used, based essentially on sequence comparison, can hardly reveal new effectors.

Recently, two groups of investigators performed large-scale screening of T4S effectors by bioinformatic analysis [17,18]. Integrating multiple features including gene G + C content, sequence conservation, within-genome gene organization, regulatory elements, signal sequence composition, etc., Burstein et al., for the first time, set up a machine learning method to predict and experimentally identify new T4S effectors from *Legionella pneumophila*[17]. The prediction accuracy was considerably high, but the method developed is merely suitable for T4S protein prediction in *Legionella* or closely-related species, since the training sequences are all from *Legionella* and the features about sequence conservation, gene organization and regulatory elements are specific for *Legionella*. In addition, a similar training pipeline is infeasible to develop T4S effector predictors for a broader range of bacteria, because the numbers of validated T4S effectors in most other bacterial genera, not like in *Legionella* (more than 100), are so small (0 ~ 5) that the training data cannot provide reliable feature information. In another study, based on the weak sequence similarity with *Legionella* effectors, Chen et al. identified a group of effectors in *Coxiella burnetii*[18]. Most effectors, especially those in the distantly-related species, however, are of no or very low sequence similarity. Therefore, new effectors without sequence similarity cannot be captured through sequence alignment.

We have focused on *Helicobacter pylori* to predict T4S effectors for insights into the pathogenesis of the distinct infections caused by these bacteria. *H. pylori* may elicit human gastritis and gastric ulcer, and this pathogen is also associated with gastric cancer [4]. In the pathogenesis, Cag-T4SS plays key roles as an important virulence factor in the bacterial interaction with human stomach cells [3,4]. To date, only one effector, CagA, has been identified, although several lines of evidence have indicated that there should be other effectors that participate in bacterial infection and pathogenesis [4,19,20]. No experimental, sequence alignment or comparative genomic methods are available for identifying new effectors. The only CagA protein could not provide any statistic information about its sequence features as a T4S effector either.

Numerous reports have indicated that, in many different bacteria, the C-terminal peptide sequences of T4S effectors are necessary for their secretion [21-25]. Do these amino acid sequences share any common composition or structural features among different effectors in different bacterial species? Could such features, if any, be used to develop an inter-species T4S effector predictor? Such a generally-suitable prediction tool would be especially useful for identification of new effectors in species like *H. pylori*, which is supposed to have multiple effectors that are not experimentally validated yet and lacks a sufficient number of within-species validated effectors for species-specific effector feature extraction. Recently, many inter-species prediction tools have been developed to predict Type III secreted (T3S) effectors [26-32], but no similar software tool has been developed for T4S effector prediction. In this research, we collected a full set of T4S effectors and made systematical comparisons of their C-terminal sequence-based and position-specific amino acid compositions, motifs, secondary structures and solvent accessibility properties. Based on these features, we developed a series of machine learning methods to classify T4S effectors and non-effectors. To our knowledge, this is the first inter-species T4S protein prediction tool, which can be applied to different bacteria and is especially useful for bacteria that have limited effector information for species-specific bioinformatic analysis.

## Results

### Sequence-based amino acid composition (Aac) differences between C-termini of T4S and non-T4S proteins

The T4S proteins were annotated from literature, while the non-T4S proteins were randomly selected from the genome-encoding proteins removed of known T4S proteins and their homologs (Methods). The size of non-T4S proteins was twice of T4S proteins. The GC content of the nucleotide sequences encoding the T4S proteins was roughly equal to that of non-T4S encoding nucleotide sequences (Methods).

**Figure 1:**
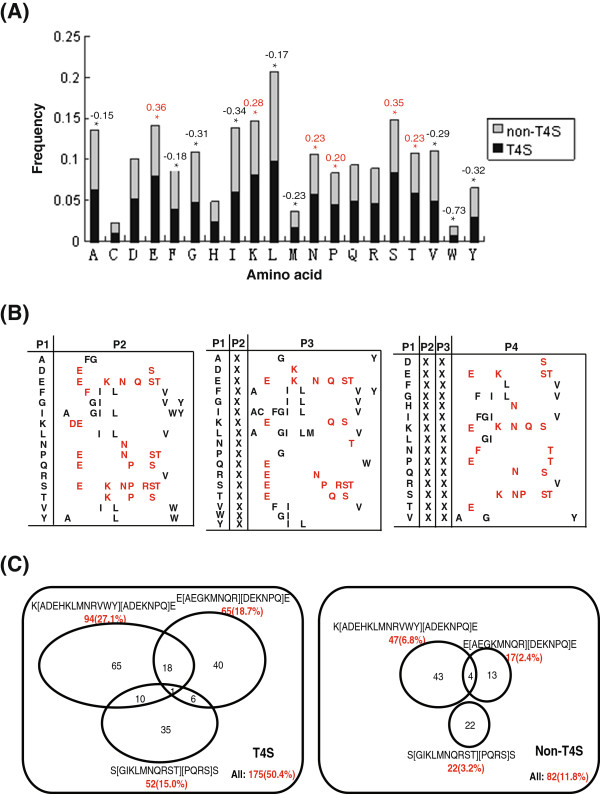
**Sequence-based Aac difference between T4S and control proteins for C-terminal 100-aa positions. (A)** Single-residue composition difference. The different amino acids were listed along the horizontal axis while the length of bars represented the frequency of the corresponding amino acid. T4S and non-T4S proteins were represented in black and gray, respectively. Amino acid with significant different compositions between effectors and non-effectors were indicated with a star above the bar (Bonferroni-corrected Student’s *t* test and binomial test, *p* < 0.05). The logarithm of amino acid frequency ratio was also shown, with red representing preference and black representing depletion in effectors. **(B)** Continual and spanned bi-residues with statistically significant composition difference between effectors and non-effectors (Bonferroni-corrected Student’s *t* test and binomial test, *p* < 0.05). ‘Px’ represented ‘Position x’. ‘X’ represented any type of amino acid. The amino acid at the last position was in red if the corresponding bi-residue was preferred and in black if depleted in T4S sequences. **(C)** Distribution of motifs in T4S and non-T4S proteins.

Comparisons were performed on sequence-based Aac of C-terminal 100 positions (C100) between T4S and non-T4S sequences. Most amino acid species were not equally distributed in the two types of sequences, with glutamic acid, serine, lysine, threonine, asparagine and proline enriched and isoleucine, glycine, valine, tyrosine, tryptophan, methionine, leucine, phenylalanine and alanine depleted in T4S sequences (*p* < 0.05, Bonferroni-corrected binomial test and *t*-test; Figure 1A). The relative enrichment ratio of Aac was calculated for each amino acid species which showed statistical difference between T4S and non-T4S sequences. Glutamic acid and serine had the largest enrichment in T4S sequences, whereas isoleucine, tyrosine and glycine were enriched in non-T4S sequences (tryptophan and methionine were not considered because of their low occurrence in both types of sequences) (Figure 1A). The relative enrichment ratios of biased amino acids between T4S and non-T4S sequences were not apparently enlarged when the length of observed signal sequence was shortened (Additional file 1: Figure S1).

Bi-residue composition (bAac) was also compared between T4S and non-T4S C100 sequences. Among the 400 combinations of bi-residues, 29 were significantly enriched and 25 were depleted in T4S sequences (*p* < 0.05, Bonferroni-corrected binomial test; Figure 1B left and Additional file 2: Table S1). Most significantly enriched bi-residues included ‘[EKD]E’, ‘[STPE]S’, ‘E[TKN]’, ‘S[PE]’, ‘FF’, ‘TP’ and ‘PT’, while ‘I[IAG]’, ‘[VYF]L’, ‘G[IVG]’ and ‘[FV]I’ were most significantly depleted in T4S sequences (‘[XY]’ means ‘X’ or ‘Y’; Additional file 2: Table S1). The composition was further compared for discontinuous bi-residues (eg., ‘ExxS’, ‘ExSx’, etc., where targeted bi-residues ‘E’ and ‘S’ were interupted by other residues). Twenty two bi-residues interrupted by one amino acid were enriched and 31 were depleted in T4S sequences, among which ‘[KE]XE’, ‘[ESTK]XS’, ‘EX[TK]’ and ‘SX[PER]’ were most significantly enriched while ‘[GLVI]XI’, ‘[IGL]XV’, ‘AXY’, ‘LXA’ and ‘[YI]XL’ were most significantly depleted (‘X’ represents any amino acid; Figure 1B middle and Additional file 2: Table S1). Among the bi-residues interrupted by two amino acids, ‘[EKP]XXE’, ‘SXX[SKTPN]’, ‘EXX[TK]’, ‘NXXT’ and ‘DXXS’ were most significantly enriched, and ‘[GI]XXI’, ‘IXX[GF]’, ‘GXX[FL]’, ‘VXX[AG]’ and ‘LXXG’ were most significantly depleted in T4S sequences (Figure 1B right and Additional file 2: Table S1). Among these continuous and interrupted bi-residues, ‘KXXE’, ‘SXXS’ and ‘EXXE’ existed in 193 (56%), 187 (54%) and 183 (53%) T4S sequences, respectively, representing the patterns most enriched in T4S signal peptides. Nearly 90% (312/347) of the T4S sequences contained at least one of the three motifs. However, the percentages of non-T4S sequences containing such motifs were much lower (34%, 30% and 31% for ‘KXXE’, ‘SXXS’ and ‘EXXE’ respectively, and 67% for existence of at least one of the three motifs).

Tri-residue (tAac) and quart-residue (qAac) compositions were further compared, so as to refine the conserved motifs buried in T4S signal sequences. Taking into account of the bi-residue composition preference property described above, an consensus method disclosed three degenerate motifs, ‘K[ADEHKLMNRVWY][ADEKNPQ]E’, ‘E[AEGKMNQR][DEKNPQ]E’, and ‘S[GIKLMNQRST][PQRS]S’, which were significantly enriched in T4S sequences (*p* < 0.05, Bonferroni-corrected binomial test). In total, more than 50% (175/347) of the T4S sequences contained at least one of these three motifs, whereas only 12% (82/694) of the non-T4S sequences contained one or more of them (Figure 1C and Additional file 3: Table S2). The motifs existed in effectors of different bacteria with IVA or IVB T4SS (Additional file 3: Table S2).

The patterns with more than four residues were quite degenerate, and represented by very few T4S sequences (data not shown).

### Distinct position-specific Aac profiles in C-termini of T4S effectors

**Figure 2:**
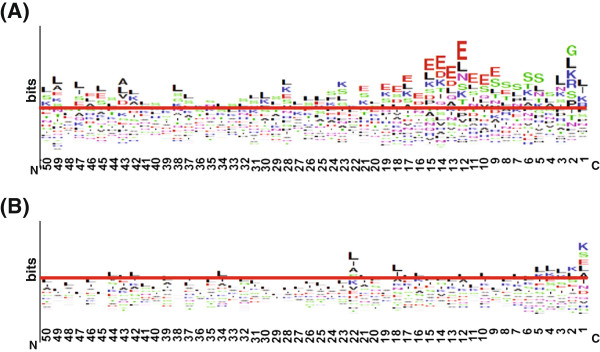
**Position-specific Aac profiles of T4S and control proteins for C-terminal 50 positions.** The horizontal axis indicates the C-terminal position number. **(A)** and **(B)** represent T4S proteins and control proteins, respectively.

Besides sequence-based Aac preference in T4S signal peptides, the position-specific Aac profiles were also compared between T4S and non-T4S sequences. As shown in Additional file 4: Figure S2 and Figure 2, T4S sequences showed apparently different amino acid composition profiles from non-T4S sequences. These differences were most striking for C-terminal 1–50 (especially 1–25) positions (Additional file 4: Figure S2). More positions in T4S effectors exhibited specific amino acid preference, while in non-T4S sequences, different species of amino acids appeared more evenly distributed at each position (Figure 2A and B). Consistent with the sequence-based observations, glutamic acid, serine and lysine were also frequently preferred in T4S sequences (Figure 2A). Leucine was enriched in both T4S and non-T4S sequences (Figure 2A and B).

**Figure 3:**
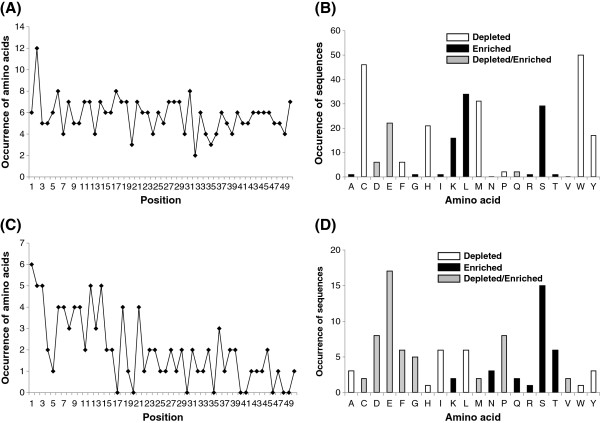
**Distribution of amino acids with significant different position-specific composition. (A)** and **(B)** show the distribution of significantly preferred or unfavorable amino acids in T4S proteins, respectively. **(C)** and **(D)** show the distribution of amino acid with significantly different composition between T4S and control proteins. **(A)** and **(C)** compare the numbers of significantly different amino acids at each position. **(B)** and **(D)** showed the times of each type of amino acid exhibiting significant difference.

To further evaluate whether the observed amino acid preference (or depletion) is statistically significant, we set up a binomial distribution model for each amino acid at each position of T4S and non-T4S C-terminal 50 positions. At positions of T4S C-termini, the 20 amino acid species did not show equal preference. Some amino acids were enriched while some others depleted significantly (Figure 3A; Additional file 5: Table S3). Tryptophan and cysteine were most generally depleted in T4S C-termini. Additionally, leucine (enriched), methionine (depleted), serine (enriched), glutamic acid (enriched or depleted) and histidine (depleted) were also frequently biased in the composition (Figure 3B; Additional file 5: Table S3). The total number of amino acids with significant position-specific composition difference between T4S and non-T4S proteins was much smaller than that of theoretically biased amino acids in T4S proteins, demonstrating that there are many common amino acid composition biases between the two types of proteins (Additional file 5: Table S3). However, the difference between T4S and non-T4S proteins was even more pronounced at the C-terminal 30 positions (Figure 3C). The most profound composition difference between T4S and non-T4S in most positions was the frequency bias of glutamic acid (enriched or depleted), followed by those of serine (enriched), aspartic acid (enriched or depleted), proline (enriched or depleted), threonine (enriched) and phenylalanine (enriched or depleted) (Figure 3D). It should be noted that, leucine was also frequently biased (depleted) in T4S sequences compared with its composition in non-T4S sequences, indicating the larger enrichment in the latter (Figure 3B and D). Other amino acids, e.g., cysteine, tryptophan, methionine and histidine, did not contribute much to the composition bias, as they are depleted in both T4S and non-T4S proteins (Figure 3B and D). Notably, glutamic acid, though enriched in most C-terminal positions of T4S proteins when compared with non-T4S proteins, showed significant depletion in C-terminal 1–4 positions of T4S proteins and was significantly enriched at positions 9 to 19 continuously (Additional file 5: Table S3). Some of the amino acids enriched or depleted in T4S sequences (e.g., serine, threonine, proline and glutamic acid) could be related with the secondary structure and hydrophilicity, two possibly important secondary features related with signal recognition [26,30]. The biological relevance of the biases of the amino acids remains to be clarified.

### Structural flexibility of the C-termini of T4S effectors

The primary peptide sequence determines its secondary structure (Sse) and solvent accessibility (Acc), which may be associated with the specificity of signal recognition. Therefore, we compared the Sse and Acc composition in each C-terminal position of T4S effectors with those of the non-T4S proteins. As expected, T4S effectors showed a position-specific Sse preference pattern apparently different from that of the non-T4S proteins in the C-terminal region, especially at the C-terminal 40 positions (Additional file 6: Figure S3A and B). In contrast to helices in the non-T4S sequences, coils are more common in most regions of the T4S sequences, indicating that they are more flexible (Additional file 6: Figure S3A and B). Besides, β-strands were less frequently adopted by T4S sequences (Additional file 6: Figure S3A and B). T4S and non-T4S sequences also showed different position-specific Acc profiles, with more positions being exposed in the C-termini of T4S sequences (Additional file 6: Figure S3C and D). The distinct Sse and Acc profiles adopted by the C-terminal region of T4S effectors were similar to those of N-terminal region of type III secreted (T3S) proteins, indicating possibly similar signal recognition mechanisms between the type IV and type III secretion systems [26].

When twenty T4S C-terminal peptides were randomly selected for 3D structure prediction, six peptides were predicted with high accuracy. The C-terminal ends of all the six peptides form helices or coiled coils, always exposed outside (Additional file 7: Figure S4). A structure alignment showed that these six peptides could form a cluster with quite similar structures (37% structure similarity, <10 Å; Additional file 8: Figure S5A). Most interestingly, though without similarity at the sequence level, *Legionella* VipE (YP_096808.1) and YP_094180.1 had an extremely similar 3D structure, with a mirror symmetry for the C-terminal end parts (76% structure similarity, <5 Å; Additional file 8: Figure S5B). *Legionella* YP_094076.1 and *Coxiella* YP_001597263.1 also showed 74% similarity, and these four proteins, VipE, YP_094180.1, YP_094076.1 and *Coxiella* YP_001597263.1, had 52% structure similarity (<10 Å; Additional file 8: Figure S5C and D). The 3D structure similarity suggested that the high-order structure could exert important function in specific T4S signal recognition.

### Inter-species prediction of T4S effectors based on Aac and structural features

It is interesting to determine whether the distinct Aac (sequence-based and position-specific), motifs, Sse and Acc profiles can be used for distinguishing T4S proteins. Support Vector Machine (SVM) based machine learning models were therefore trained with different features and/or their combination, and comparison was performed on their classification power. SVM was adopted since it often generates high classification accuracy and especially high specificity [26-28,31]. Additional file 9: Table S4 showed the parameters optimized for different models.

**Figure 4:**
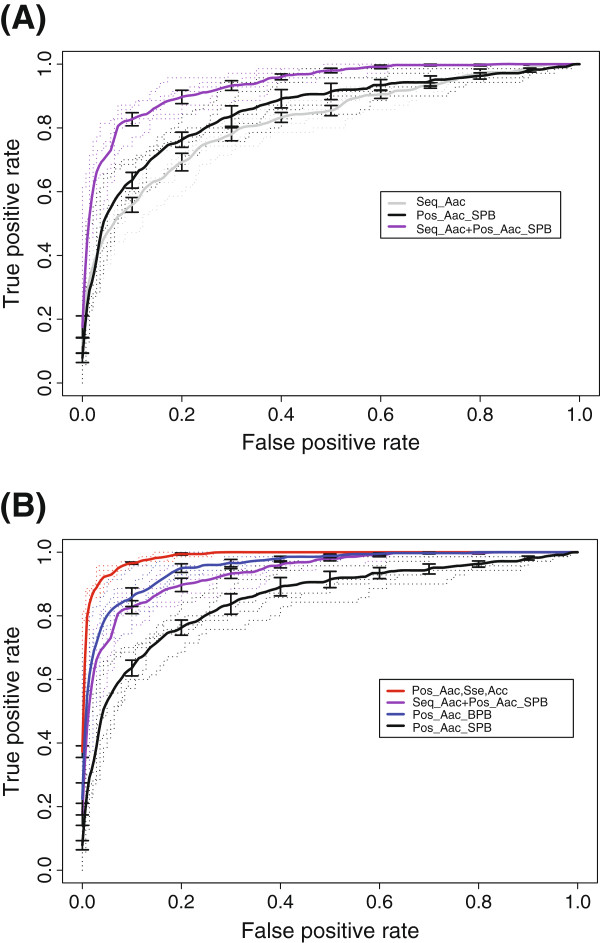
**Performance ROCs of different T4S effector prediction models. (A)** Comparison of ‘Pos_Aac_SPB’, ‘Seq_Aac’, and ‘Pos_Aac_SPB + Seq_Aac’ models. ‘Pos_Aac_SPB’ only extracted the features of positive dataset. ‘Seq_Aac’ only learned sequence-based single-residue composition features. ‘Pos_Aac_SPB + Seq_Aac’ combined the features of ‘Pos_Aac_SPB’ and ‘Seq_Aac’. **(B)** Comparison of ‘Pos_Aac_SPB’, ‘Pos_Aac_BPB’, ‘Pos_Aac_SPB + Seq_Aac’ and ‘Pos_Aac,Sse,Acc’ models. ‘Pos_Aac_BPB’ model extracted the Aac features of both positive and negative datasets, while ‘Pos_Aac,Sse,Acc’ learned the joint position-specific Aac, Sse and Acc features. All comparisons were performed with a 5-fold cross-validation strategy.

As shown in Table 1, the decision model based only on motifs detected above had the worst distinguishing power, with an average accuracy of 75.6%. The distinguishing power was similar among the models based on sequential Aac, bi_residue composition (bAac), their combination and the combination of significantly biased Aac and bi_Aac between T4S and non-T4S peptides, in terms of sensitivity, specificity, accuracy, AUC and MCC values (Table 1). The SVM model based on position-specific, single-profile bayesian (SPB) features only performed a little better than the sequence-based models (Table 1). The Bi-Profile Bayesian (BPB) model, however, considerably outperformed both the SPB model and the sequence-based models (Table 1 and Figure 4A). Interestingly, the combination of SPB Aac features and sequential Aac features could greatly improve the classifying performance, which was comparable to that of BPB Aac model (Table 1 and Figure 4A).

**Table 1:** Performance of different models classifying T4S effectors and non-effectors

**Features**	**Model**	***Sn *** **(%) vs. *Sp *** **(%)**	***A *** **(%)**	***AUC***	***MCC***
Seq_Aac	SVM	50.57 vs. 93.86	79.43	0.8212	0.5146
Seq_bAac	SVM	44.57 vs. 96.29	79.05	0.8311	0.5088
Seq_Aac, bAac	SVM	46.00 vs. 96.14	79.43	0.8343	0.5182
Seq_Sig	SVM	50.57 vs. 93.86	79.43	0.8500	0.5146
Motif	-	50.43 vs. 88.18	75.60	-	0.4222
Seq_Aac, Sse, Acc	SVM	69.71 vs. 91.14	84.00	0.8742	0.6313
Pos_Aac_SPB	SVM	61.71 vs. 92.14	82.00	0.8538	0.5802
Pos_Aac _SPB + Seq_Aac	SVM	78.86 vs. 93.29	88.48	0.9362	0.7369
Pos_Aac_BPB	BPB-SVM	79.14 vs. 94.43	89.33	0.9559	0.7561
Pos_Aac, Sse, Acc	BPB-SVM	89.14 vs. 97.14	94.57	0.9883	0.8770

Note: The RBF kernel function was used for all the models except ‘Motif’. The performance was evaluated according to 5-fold cross validation results.

Inclusion of secondary structure and solvent accessibility improved the distinguishing power of both sequence-based models and position-specific Bayesian models. The model based on sequential joint features of Aac, Sse and Acc outperformed any other pure sequential features-based model (Table 1). Most strikingly, the position-specific model based on the joint features outperformed all other models in terms of any evaluation parameter (Table 1 and Figure 4B). The five-fold cross-validation sensitivity, specificity, accuracy, AUC and MCC of this model could achieve 89.14%, 97.14%, 94.57%, 0.9883 and 0.8770, respectively (Table 1).

We also tested the influence of different signal sequence length on model performance. Among the models based on C-terminal 25aa, 30aa, 40aa, 50aa and 100aa (C25, C30, C40, C50 and C100, respectively), C100 models apparently outperformed the others (data not shown). Since the models based on combined SPB Aac and sequential Aac features (T4SEpre_psAac), BPB Aac features (T4SEpre_bpbAac) and position-specific joint features of Aac, Sse and Acc (T4SEpre_Joint) showed the best performance on classification of T4S and non-T4S sequences, the rest parts of the research will only use these three models based on C-terminal 100aa signals. To further confirm the classification performance of these three models, we changed the size of negative dataset (from 2-fold to 6-fold size of the positive dataset, Additional file 10: Text S1), and assessed the performance with 5-fold and 10-fold cross validation. As shown in Additional file 11: Table S5 and Additional file 12: Table S6, the prediction performance was improved slightly when the negative dataset with larger size (Additional file 11: Table S5) was used and quite stable when 5-fold (Additional file 11: Table S5) or 10-fold (Additional file 12: Table S6) cross validation was adopted.

**Figure 5:**
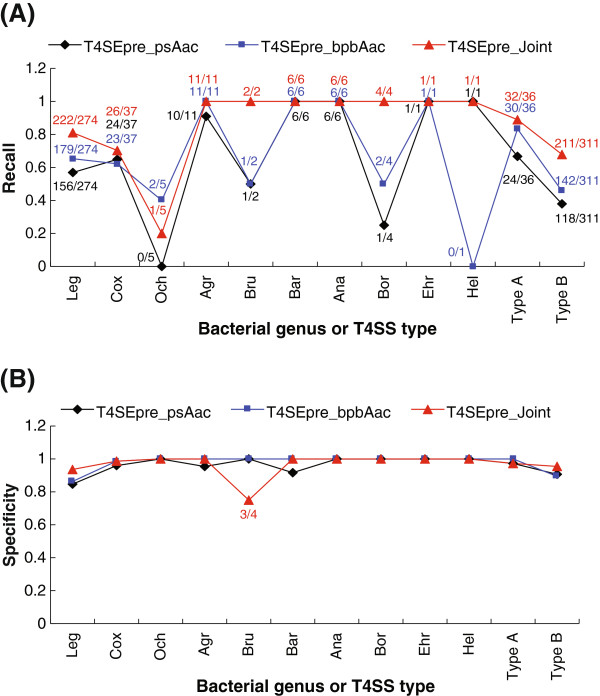
**Inter-species/group prediction of T4S effectors by three computational models with a Leave-One genus-Out strategy. (A)** Recall of known effectors in each species or group. Agr, Ana, Bar, Bor, Bru, Cox, Ehr, Hel, Leg and Och represented *Agrobacterium*, *Anaplasma*, *Bartonella*, *Bordetella*, *Brucella*, *Coxiella*, *Ehrlichia*, *Helicobacter*, *Legionella*, and *Ochrobactrum* respectively. Type A and B represented the two types of T4SSs. **(B)** Prediction specificity of different models in each species or group.

It is also important to observe the inter-species effector discriminating power of the models. A Leave-One genus-Out strategy was proposed previously and adopted here. As shown in Figure 5, T4SEpre_Joint exhibited the best inter-species prediction performance, while T4SEpre_psAac performed worst among the three software tools. For most genera, T4SEpre_Joint could recall all or nearly all known effectors without any prior knowledge about the targeted genus (Figure 5A) and at very high prediction specificity (Figure 5B). The specificity of T4SEpre_Joint for *Brucella* appeared lower because the total number of negative control proteins was only 4, and in fact, merely one of them was misclassified (Figure 5B). It is worth pointing out that only 73 training effectors remained after all the 274 *Legionella* effectors were excluded, and the T4SEpre_Joint model with such limited training data (21% of the original training data) could still correctly recognize most of the known *Legionella* effectors (222/274, 81%). One genus, *Ochrobactrum,* was an apparent exception: the models based on the effectors of other genera could at best recall 2/5 of the known effectors (Figure 5A, T4SEpre_bpbAac).

There are two types of T4SSs, type A and type B. It is interesting to observe the inter-category discretion power of these models. The effectors were therefore assigned to two subsets, type A T4SS substrates and type B T4SS substrates. The negative controls were divided into two parts accordingly. Models were trained with either one type of sequences and were further used to classify the other type of sequences. As shown in Figure 5, whereas T4SEpre_bpbAac and T4SEpre_psAac also showed some performance, T4SEpre_Joint showed the best classification power. The relatively low recall rates of type B effectors (67.85% for T4SEpre_Joint) with the model based on type A effectors were due to the extremely limited number of type A effectors (36/347, 10.4%) (Figure 5A). Again, the specificity of different models on either type was very high, further demonstrating the reliability of inter-species prediction with all these three software tools (Figure 5B).

Taking together, the results demonstrated that the features purely extracted from C-terminal sequences could well distinguish T4S effectors and non-effectors. The models, especially T4SEpre_Joint, showed an excellent inter-species prediction performance.

### New T4S effector candidates in *H. Pylori* and *salmonella typhiumium*

*H. pylori* is reported to encode multiple T4S effectors [4,19,20], among which only one, CagA, has been experimentally validated. As a result, direct statistic feature analysis for *H. pylori* effectors is impossible. It has been a big challenge to look for new effectors in *H. pylori*. We therefore used T4SEpre (Additional file 13), the inter-species T4S effector prediction software containing 3 highly-efficient models (T4SEpre_Joint, T4SEpre_bpbAac, and T4SEpre_spAac), to screen the *H. pylori* genome (NC_000915) for possible T4S effectors.

T4SEpre_Joint, T4SEpre_bpbAac and T4SEpre_spAac identified 58, 78 and 37 T4S effectors respectively (Additional file 14: Table S7). In total, 25 candidates were predicted by T4SEpre_Joint and at least one other model, which composed the most potentially true effectors (Table 2). The genes encoding these effector candidates were widely scattered throughout the genome. Among these candidates, CagA was a known effector and the rest 24 were new. Motif screening showed that more than half of the candidates contained ‘K[ADEHKLMNRVWY][ADEKNPQ]E’, ‘E[AEGKMNQR][DEKNPQ]E’, or ‘S[GIKLMNQRST][PQRS]S’ (Additional file 14: Table S7; Table 2, italic). It should be noted that ~70% of the T4S candidates were hypothetical proteins with unknown function (Table 2). Previous studies have demonstrated that many proteins with unknown function were likely to function as pathogenic effectors [27]. Therefore, these proteins deserve further experimental validation analysis.

**Table 2:** **T4S effectors predicted from *H. pylori***

**Protein_Accession**	**Annotation**	**Joint**	**bpbAac**	**psAac**
*gi|15644760|ref|NP_206930.1|*	*Hypothetical protein HP0130*	√	√	√
*gi|15645482|ref|NP_207657.1|*	*Hypothetical protein HP0863*	√	√	√
*gi|15645135|ref|NP_207305.1|*	*Hypothetical protein HP0508*	√	√	√
*gi|15644973|ref|NP_207143.1|*	*Hypothetical protein HP0345*	√	√	
*gi|15645339|ref|NP_207511.1|*	*DNA polymerase III subunits gamma and tau*	√	√	√
gi|15645728|ref|NP_207905.1|	Excinuclease ABC subunit B	√	√	
*gi|15646132|ref|NP_208314.1|*	*Hypothetical protein HP1524*	√	√	
*gi|15645173|ref|NP_207343.1|*	*Cag pathogenicity island protein (cag26,* ***cagA****)*	√	√	√
gi|15644995|ref|NP_207165.1|	Hypothetical protein HP0367	√	√	
*gi|15645343|ref|NP_207515.1|*	*Hypothetical protein HP0721*	√	√	
gi|15645618|ref|NP_207794.1|	Hypothetical protein HP1003	√	√	√
gi|15645567|ref|NP_207743.1|	Putative recombination protein RecO	√		√
*gi|15645998|ref|NP_208179.1|*	*Hypothetical protein HP1388*	√	√	
*gi|15644810|ref|NP_206980.1|*	*Hypothetical protein HP0181*	√	√	
*gi|15644793|ref|NP_206963.1|*	*Signal-transducing protein, histidine kinase*	√	√	√
gi|15645647|ref|NP_207823.1|	Hypothetical protein HP1033	√	√	
gi|15644672|ref|NP_206842.1|	Hypothetical protein HP0041	√	√	√
*gi|15644966|ref|NP_207136.1|*	*Hypothetical protein HP0338*	√	√	√
gi|15646203|ref|NP_208145.1|	Hypothetical protein HP1353	√	√	
gi|15645609|ref|NP_207785.1|	Hypothetical protein HP0994	√	√	√
gi|15644860|ref|NP_207030.1|	Hypothetical protein HP0232	√	√	√
*gi|15645621|ref|NP_207797.1|*	*Conjugal transfer protein (traG)*	√	√	
gi|15645351|ref|NP_207525.1|	Hypothetical protein HP0731	√	√	
gi|15645518|ref|NP_207693.1|	Hydrogenase expression/formation protein (hypB)	√	√	√
*gi|15645888|ref|NP_208066.1|*	*Paralysed flagella protein (pflA)*	√	√	

Note: ‘Joint’, ‘bpbAac’ and ‘psAac’ represent ‘T4SEpre_Joint’, ‘T4SEpre_bpbAac’ and ‘T4SEpre_psAac’ model, respectively. The genes with one or more of the three motifs identified in this study were in italic.

As a control, we also made a whole-genome T4S effector prediction from *Salmonella typhimurium* LT2, a strain which has never been reported with a functional protein-transporting T4SS. As shown in Additional file 15: Table S8, T4SEpre_Joint, T4SEpre_bpbAac and T4SEpre_spAac identified 57, 81 and 27 T4S effectors respectively. Dividing by the total number of genome-encoding proteins (*S. tyhimurium* LT2, 4423; *H. pylori,* 1573), the percentages of positive T4S proteins predicted in *S. tyhimurium* (1.29, 1.83 and 0.61, respectively) were lower than in *H. pylori* (3.69, 4.96 and 2.35, respectively). Furthermore, the prediction results of the three software tools were combined to increase prediction specificity, as performed in *H. pylori*. We found only 13 proteins were predicted by both T4SEpre_Joint and at least one other software tool (Additional file 15: Table S8). This positive ratio (0.29%, 13/4423) was also much lower than that in *H,pylori* (1.59%, 25/1573). Similar to the distribution of T3S signals among different bacteria, it is not surprising to find T4S signal containing proteins in strains without protein-transporting T4SSs such as *S. typhimurium* LT2, though the number of positive proteins could be much smaller [27,29,30]. Three proteins in LT2 predicted to be positive T4S effectors by all the three tools meanwhile (STM1870, STM2074 and STM2256; Additional file 15: Table S8). Among them, STM1870 is particularly interesting. It was predicted by all the three models with the highest scores and hence most likely represents a true T4S effector (Additional file 15: Table S8). In a previous report, STM1870 was also found to contain a T3S signal [27]. The function of STM1870 remains to be clarified. STM2074 is annotated as a histidinol phosphatase and STM2256 encodes a cytochrome c-type subunit. These two proteins are more likely to represent false positives predicted by the software tools, but the possibility could not be excluded either that, they contain the T4S signal sequences and could be translocated through the T4SS conduit to host cells if there was a functional T4SS in *Salmonella*.

## Discussion and conclusion

Bacteria encode diverse protein secretion or translocation systems to effectively interact with host cells. Type III and type IV secretion systems play especially important roles in gram-negative bacteria [2,9,33,34]. Through comparative genomic analysis, Guglielmini et al. found more bacteria than expected could encode potential protein-exporting T4SSs [9]. This is an interesting finding, indicating that these bacteria potentially interact with host cells by injecting effector proteins through T4SSs. It is much easier to detect whether these T4SSs are assembled and functional than to analyze how they could function. Identifying possible effectors is the determinant step to solve the latter problem. Currently, the most effective way to identify new T4S effectors is to validate candidates predicted according to the common features of known effectors encoded by the same or closely-related bacteria [17,18]. However, for most species that have T4SSs, only a small number of effectors have been identified to date. Due to the small sample pool of known T4S effectors, no reliable features could be generalized from them. The species-specific methods described above therefore could not be adopted directly either. The number of newly discovered effectors is increasing for a limited number of representative species, e.g., *L. pneumophila*, but very few new effectors are being identified for other important species, e.g., *H. pylori*. These factors prompted us to develop an inter-species T4S effector prediction method.

In this study, we focused on sequence and structure-derived features. Through sequence-based single-, bi-, tri-residue Aac and motif analysis, we found distinct composition preference in C-terminal sequences of T4S effectors relative to control proteins. Glutamic acid and serine were most strikingly preferred by T4S effector sequences (Figure 1A, B and C). Position-specific Aac comparison demonstrated significant biases in the composition of glutamic acid and serine in a number of positions. Unlike serine, which always showed preference in T4S sequences, glutamic acid was preferred in most positions but depleted in C-terminal positions 1–4 (Figure 3). In the C-terminal sequences of more than 50% effectors, three possible motifs were identified, which always contained one (or more) glutamic acid or serine as the consensus residue(s) (Figure 1C). It is interesting to examine whether and how these two amino acids or the motifs play roles in the specificity of type IV secretion recognition. The biological meaning of other Aac preference also remains to be clarified.

We also tried to observe the different secondary structure and solvent accessibility determined by the different Aac features between T4S and control proteins. The T4S effectors had much more flexible and exposed C-terminal regions than the control proteins (Additional file 6: Figure S3). We had similar observation for the N-terminal sequences of type III secreted effectors reported previously [26]. It is not clear whether this is a common property of protein secretion signal sequences. Interestingly, 3D structure modeling revealed similar tertiary structure of the T4S C-terminal sequences (Additional file 8: Figure S5). Due to the relatively low accuracy and heavy computation cost of *de novo* structure prediction, it is not feasible to predict the structure of all T4S effectors with high precision. However, it is still interesting to observe the structure basis of specific type IV secretion recognition.

A variety of computational models were trained based on the different types or combinations of features. Three of them, T4SEpre_Joint trained on joint features of position-specific Aac, Sse and Acc, T4SEpre_bpbAac trained on Bi-Profile Bayesian Aac, and T4SEpre_psAac trained on both position-specific (Single-Profile Bayesian) and sequence-based Aac features, considerably outperformed the others in terms of sensitivity, specificity, accuracy, AUC and MCC (Table 1 and Figure 4). Additionally, T4SEpre_Joint also exhibited an ideal inter-species prediction power. Due to the lack of known effectors in most bacterial species, *Legionella* effectors represented the overwhelming majority of the training data (89%). Remarkably, the T4SEpre_Joint model trained on the sequences of the other species (21% of the original training data) could still correctly recall ~ 81% of the known *Legionella* effectors (Figure 5). Even with the fewer training data (type A effectors and control proteins, 10.4% of the original training data), T4SEpre_Joint could correctly recognize ~ 68% of the relatively independent type B effectors (Figure 5). Though with lower distinguishing performance than T4SEpre_Joint, T4SEpre_bpbAac and T4SEpre_psAac revealed different features of T4S effectors. These three tools, therefore, may be combined in practice for T4S effector prediction.

Prediction of Sse and Acc is relatively time-consuming for all bacterial proteins. We therefore only used T4SEpre_bpbAac and T4SEpre_psAac to screen T4S signals in all the bacteria with possible protein-delivery T4SSs [9]. We found all the bacterial chromosomes containing protein-exporting T4SSs encode possible T4S effectors. On average, up to 5% genes encode T4S effectors (data not shown). We further focused on *H. pylori*, for which all the three T4SEpre models were adopted to predict possible new effectors other than CagA. A total of 25 genes were predicted by both T4SEpre_Joint and at least one other model. Notably, nearly 70% of the predicted genes encoded hypothetical proteins with unknown functions (Table 2). Besides, many genes, especially those with higher prediction scores, contained at least one of the three types of T4S motifs. These genes and others with high prediction values provide a valuable list of effector candidates for pathogenic study of *H. pylori*.

An ideal computational model could predict all the true positive effectors (highest sensitivity) without any false positive effector (highest specificity). However, it is infeasible to develop such a perfect model. In practice, we have to make a balance between sensitivity and specificity to cope with different situations. For example, in bacteria with many known effectors such as *Legionella*, the prediction specificity has to be sacrificed to increase the sensitivity, so as to find more new effectors. However, to identify effectors from bacteria with few known effectors such as *H. pylori*, it is recommended to increase prediction specificity at a cost of sensitivity. The higher specificity will ensure the fewer false positives and the lower experimental cost. The three software tools proposed here all exhibited quite high prediction specificity (93 ~ 97%). It should be pointed out that, even with the highest cross-validation specificity 97%, ~86 false positives would be predicted from a genome encoding 2850 non-effector proteins. The sensitivity of T4SEpre_Joint is 89% at the specificity of 97%, so about 134 effectors can be correctly predicted assuming there are 150 effector proteins in the same genome. Therefore, in a genome encoding 3000 total proteins and 150 (5%) T4S effectors, T4SEpre_Joint will predict 220 candidates, 61% (134/220) among which are true positives. In order to further increase the specificity, we suggested the following two strategies as we adopted in *H. pylori* effector prediction: (1) combining all the three tools and looking for the effectors predicted by both T4SEpre_Joint and at least one other software tool, and (2) increasing the prediction threshold value to 0.5 or higher. From our observations, the true positives are more often predicted by combining multiple models, and with higher prediction scores. Therefore, both the strategies should decrease the ratio of false positives in the prediction results.

The T4S proteins were also predicted from bacteria without known protein-transporting T4SSs (e.g., *S. typhimurium* LT2, Additional file 15: Table S8). It is not unexpected that some proteins also contain T4S signals in such bacteria. Löwer and Schneider [29] and Arnold et al. [30] independently found there were T3S signals in proteins of bacteria without known Type III Secretion Systems (T3SSs). In a previous study, we also demonstrated that T3S signals could exist in proteins of gram-negative bacteria without T3SSs, gram-positive bacteria and even yeasts [27]. Being similar with T3S signals, it makes sense that some proteins in bacteria without protein-delivery T4SSs may happen to have T4S signal sequences. Strictly, a protein containing a T4S signal sequence does not necessarily represent a T4S effector. A T4S effector must have the signal sequence, be encoded in a host strain bearing a functional protein-transporting T4SS, and can be co-expressed with T4SS apparatus genes [27]. A tentative hypothesis is, however, as in *S. typhimurium* LT2, the number of total proteins with T4S signals in bacteria without protein-transporting T4SSs should be much smaller than strains with functional protein-transporting T4SSs.

## Methods

### Datasets

Experimentally validated T4S effectors were retrieved from literature and their putative orthologs were extracted from genome annotation files. In total, we analyzed 1913 effectors from 10 genera, including *Agrobacterium*, *Anaplasma*, *Bartonella*, *Bordetella*, *Brucella*, *Coxiella*, *Ehrlichia*, *Helicobacter*, *Legionella* and *Ochrobactrum*. The T4S signal peptide, i.e., the C-terminal 100-aa fragment, was extracted from each effector sequence. Pairwise alignment was performed for the 100-aa T4S signal peptides with JAligner implementing Smith-Waterman algorithm (http://jaligner.sourceforge.net/). The ratio between the similarity score of pairwise sequences and self similarity score was calculated. Conserved paralogs or orthologs were identified when a pair of sequences had an above-stated similarity score ratio higher than 0.30. For each orthologous or paralogous cluster, only one representative was selected as the training sequence. This homology-filtering procedure reduced the number of T4S peptides to 347. The non-redundant peptides constitute the positive training dataset. Non-T4S proteins were randomly selected from the same strains where the positive training sequences were originated, followed by removal of the known T4S effectors and their homologs. The C-terminal 100-aa peptide fragment was also extracted from each non-T4S protein, and the same homology-filtering procedure was performed. Finally, for each strain, the ratio of non-T4S: T4S peptides was set as 2:1, and the GC content for encoding nucleotides was generally maintained equal or similar between the two types of sequences (T4S 40% vs. Non-T4S 41%) [26]. The 347 T4S and 694 non-T4S sequences constituted final positive and negative dataset, respectively (Additional file 16: Text S2). For 5-fold (or 10-fold) cross-validation, the negative and positive training datasets were pooled as the final training dataset, which was evenly split into five (or ten for 10-fold cross-validation) sub-datasets, each containing the same number of positive/negative samples.

To observe whether the size of negative dataset influence the classifying prediction performance, another independent negative dataset was prepared (Additional file 10: Text S1). The proteins were randomly selected from different bacteria (from all the bacteria classes listed in NCBI Genome database). The C-terminal 100 amino acids were extracted from each protein, and then a similar homology-filtering strategy was performed to get rid of the known effector homologs and redundant homologs of included negative sequences. Finally, 2082 non-redundant negative sequences were included (6-fold size of the positive dataset). These negative sequences were combined with the positive T4S sequences to form an independent training dataset. For the new sequences, Sse and Acc were predicted with the same procedures described before.

### Extraction of sequence-based and position-specific Aac features

Sequence-based Aac was calculated for each T4S or non-T4S sequence. Each of the 20 amino acid species was counted for its occurrence within the C-terminal 100, 50 and 30 positions (C100, C50, and C30 respectively). An Aac frequency vector was obtained for each sequence, and the vectors for all sequences composed a frequency matrix. The composition of each amino acid species was compared between T4S and non-T4S sequences with Student’s two-tail *t*-test and a binomial distribution-based statistic test. The resulted *p*-value was further adjusted by Bonferroni multiple testing correction [35]. The significance level was set as *p* < 0.05 for both tests. For each amino acid species with significant bias, the log ratio of average composition was calculated between the two types of sequences, which represented the relative advantage of the amino acid composition in T4S (positive) or non-T4S (negative) sequences, with a larger absolute value for a more striking advantage. The bi-residue (bAac) and tri-residue (tAac) compositions were calculated with a similar procedure. Putative and conserved motifs were screened with MEME [36], followed by an iterative calculation of the frequency of possible motifs derived from single Aac, bAac or tAac preference.

The position-specific Aac features were extracted as follows. Let vector *S* = *s*_1_, *s*_2_, *s*_3_,…, *s*_*n*_ denote a peptide sequence in which *s* represents amino acid while 1, 2,… or *i* represents position and *n* represents sequence length. For *m* sequences, the position-specific occurrence of a certain amino acid A is described as: *p*(A_*i*_) = *f* (A_*i*_)/*m*_*i*_, in which *f* (A_*i*_) denotes the frequency of amino acid A at position *i*. For each position, the *p*(A_*i*_) of different amino acids form a position set, and for a sequence *S* with *n* amino acids, *n* values (extracted from each position set) comprise a composition vector. A binomial distribution B_*i*_(*m, p*_*aa*_) was modeled for each amino acid species at each position, where *p*_*aa*_ was set as p(A_*i*_) of negative dataset or 1/20 (ideal random situation) for different comparison purpose. A Bonferroni-corrected binomial test was performed based on the distribution model to find out the significantly preferred or un-favored amino acids at corresponding position of T4S sequences. The significance level was also set as *p* < 0.05.

### Secondary structure, solvent accessibility and tertiary structure

SCRATCH was used to predict the secondary structure (Sse, represented as a combination sequence of ‘C’, ‘H’ or ‘E’ of each sequence where ‘C’ meant coil, ‘H’ meant helix and ‘E’ meant strand) and solvent accessibility (Acc, a combination of ‘b’ or ‘e’, representing ‘buried’ or ‘exposed’ respectively) [37]. Tertiary structure of T4S peptides were predicted with I-TASSER [38]. The structures with TM-score ≥ 0.5 were further analyzed for their structural similarity using MultiProt [39].

### Models and performance assessment

Sequence-based Aac features were directly represented by the frequency of each amino acid species (‘Seq_Aac’) or each bi-residue (‘Seq_bAac’). The combination of all the ‘Seq_Aac’ and ‘Seq_bAac’ features or those significantly preferred/depleted in T4S peptides led to the features of model ‘Seq_Aac, bAac’ or ‘Seq_Sig’, respectively. The sequence-based joint Aac, Sse and Acc features were extracted with the strategy described in Yang et al., [28]. Position-specific Single-Profile and Bi-Profile Bayesian features were extracted with the same pipeline for the type III secreted effector prediction model BPBAac [26]. The combination of sequence-based Aac and position-specific Single-Profile Aac features formed the features of model ‘Pos_Aac _SPB + Seq_Aac’. Position-specific joint Aac, Sse and Acc features were extracted according to Wang et al., [27]. The feature values for each training sequence formed a vector. The vectors were further trained with an R package ‘e1071’ implementing SVM (http://cran.r-project.org), with radial basis kernel function. The parameters for SVM were optimized using grid search based on 10-fold cross-validation.

The model performance was evaluated and compared with a five-fold cross-validation and Leave-One genus-Out strategy [26]. Accuracy (*A*), Specificity (*Sp*), Sensitivity (*Sn*), Receiver Operating Characteristic (ROC) curve, the area under *ROC* curve (*AUC*) and Matthews Correlation Coefficient (*MCC*) were utilized to assess the predictive performance. In the following formula, *A* denotes the percentage of both positive instances (T4S) and negative instances (non-T4S) correctly predicted. *Sn* (true positive rate) and *Sp* (true negative rate), respectively, represent the percentage of positive instances (T4S) and the percentage of negative instances (non-T4S) correctly predicted. An ROC curve is a plot of *Sn* versus (1 - *Sp*) and is generated by shifting the decision threshold. *AUC* gives a measure of classifier performance. *MCC* takes into account true and false positives and false negatives and is generally regarded as a balanced measure which can be used even if the classes are of very different sizes.

$$\begin{array}{c} A=\frac{\mathrm{TP}+\mathrm{TN}}{\mathrm{TP}+\mathrm{FP}+\mathrm{TN}+\mathrm{FN}},S_{P}=\frac{\mathrm{TN}}{\mathrm{TN}+\mathrm{FP}},S_{n}=\frac{\mathrm{TP}}{\mathrm{TP}+\mathrm{FN}},\\ \mathrm{MCC}=\frac{\left(\mathrm{TP}\times \mathrm{TN}\right)‒\left(\mathrm{FN}\times \mathrm{FP}\right)}{\sqrt{\left(\mathrm{TP}+\mathrm{FN}\right)\times \left(\mathrm{TN}+\mathrm{FP}\right)\times \left(\mathrm{TP}+\mathrm{FP}\right)\times \left(\mathrm{TN}+\mathrm{FN}\right)}} \end{array}$$

where, and denote the number of true positives, true negatives, false positives and false negatives, respectively.

### Genome-wide prediction of T4S effectors

The proteins were deduced from the *H. pylori* genome (NC_000915) and *S. typhimurium* LT2 (NC_003197) DNA sequences downloaded from the NCBI Genome database. The sequences were screened for possible T4S effectors with three independent models in the T4SEpre package (T4SEpre_Joint, T4SEpre_bpbAac and T4SEpre_psAac). The default cutoff SVM scores (≥ 0.5) were adopted for all the three models. The standalone T4SEpre package could be freely downloaded from the web site: http://biocomputer.bio.cuhk.edu.hk/softwares/T4SEpre/.

## Competing interests

The authors declare that they have no competing interests.

## Authors’ contributions

YW and SLL conceived and designed the project; YW and XW annotated T4S effectors and control proteins; YW, XW and HB analyzed the data; YW developed the models and wrote the software; YW and SLL wrote the manuscript. All authors read and approved the final manuscript.

## Supplementary Material

Additional file 1: Figure S1 Logarithm of Aac ratios between T4S and non-T4S C-terminal sequences. The different amino acids were listed along the horizontal axis while the length of bars represented the logarithm of composition ratio of the corresponding amino acid. Three lengths of T4S and non-T4S C-terminal sequences were analyzed, with C100, C50 and C30 representing C-terminal 100-aa, 50-aa and 30-aa peptides, respectively. Click here for file

Additional file 2: Table S1 Significantly biased sequential bi-Aac. Click here for file

Additional file 3: Table S2 Motifs in signal sequences of T4S effectors. Click here for file

Additional file 4: Figure S2 Position-specific Aac profiles of T4S and control proteins for C-terminal 100 positions. The horizontal axis indicates the C-terminal position number. **(A)** and **(B)** represent T4S proteins and control proteins, respectively. Click here for file

Additional file 5: Table S3 Significantly biased position-specific Aac. Click here for file

Additional file 6: Figure S3 Position-specific Sse and Acc profiles of T4S and control proteins for C-terminal 100 positions. The horizontal axis indicates the C-terminal position number. **(A)** and **(B)** represent the Sse of T4S proteins and control proteins, respectively. **(C)** and **(D)** represent the Acc of T4S proteins and control proteins, respectively. Click here for file

Additional file 7: Figure S4 3D structure of C-terminal 100aa peptides of T4S effectors. **(A)***Legionella* VipE; **(B)***Legionella* YP_094180.1; **(C)***Legionella* YP_094076.1; **(D)***Coxiella* YP_001597263.1; **(E)***Legionella* YP_094096.1; **(F)***Legionella* YP_094157.1. Click here for file

Additional file 8: Figure S5 Structural similarity among C-termini of T4S effectors. **(A)** The structure cluster formed by all the six T4S effectors with high prediction accuracy (*Legionella* VipE, YP_094180.1, YP_094076.1, YP_094096.1, YP_094157.1 and *Coxiella* YP_001597263.1); **(B)** Structure alignment between *Legionella* VipE and YP_094180.1; **(C)** Structure alignment between *Legionella* YP_094076.1 and *Coxiella* YP_001597263.1; **(D)** Structure alignment among *Legionella* VipE, YP_094180.1, YP_094076.1 and *Coxiella* YP_001597263.1. Click here for file

Additional file 9: Table S4 Optimized parameters for different SVM models classifying T4S effectors and control proteins. Click here for file

Additional file 10: Text S1 6-fold negative dataset. Click here for file

Additional file 11: Table S5 Performance of models classifying T4S effectors and non-effectors (data size ratio between negative and positive data: 6:1; 5-fold cross validation). Click here for file

Additional file 12: Table S6 Performance of models classifying T4S effectors and non-effectors (data size ratio between negative and positive data: 6:1; 10-fold cross validation). Click here for file

Additional file 13 T4SEpre package. Click here for file

Additional file 14: Table S7 T4SEs predicted from *H*. *pylori*. Click here for file

Additional file 15: Table S8 T4SEs predicted from *S*. *typhimurium* LT2. Click here for file

Additional file 16: Text S2 Training datasets. Click here for file
