# Supplementary figures and images for: Prediction of bacterial type IV secreted effectors by C-terminal features
Source: BMC Genomics. 2014 Jan 21;15:50. doi: 10.1186/1471-2164-15-50 (PMC3915618; doi:10.1186/1471-2164-15-50)

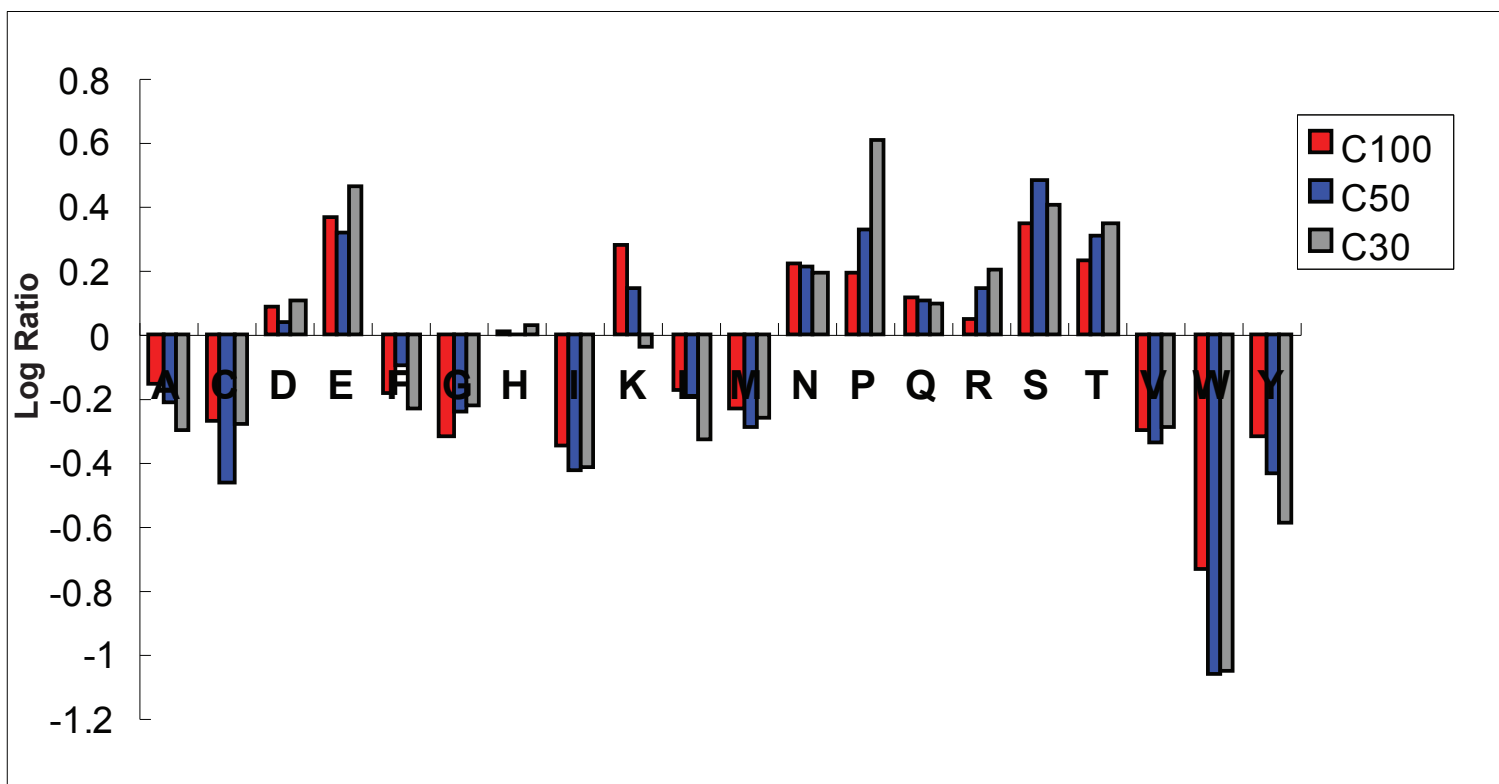

Supplement: Additional file 1: Figure S1 — Logarithm of Aac ratios between T4S and non-T4S C-terminal sequences. The different amino acids were listed along the horizontal axis while the length of bars represented the logarithm of composition ratio of the corresponding amino acid. Three lengths of T4S and non-T4S C-terminal sequences were analyzed, with C100, C50 and C30 representing C-terminal 100-aa, 50-aa and 30-aa peptides, respectively. [file 1471-2164-15-50-S1.pdf]

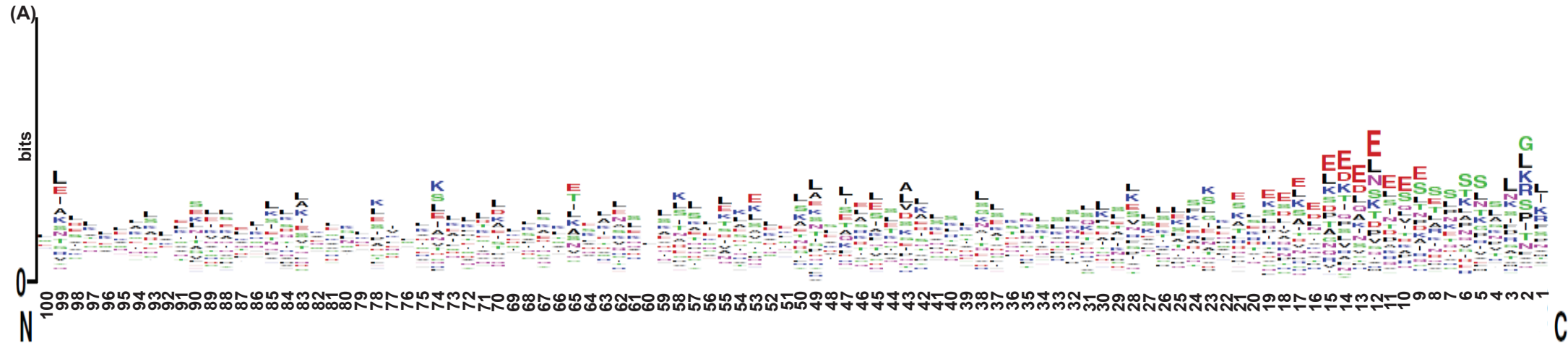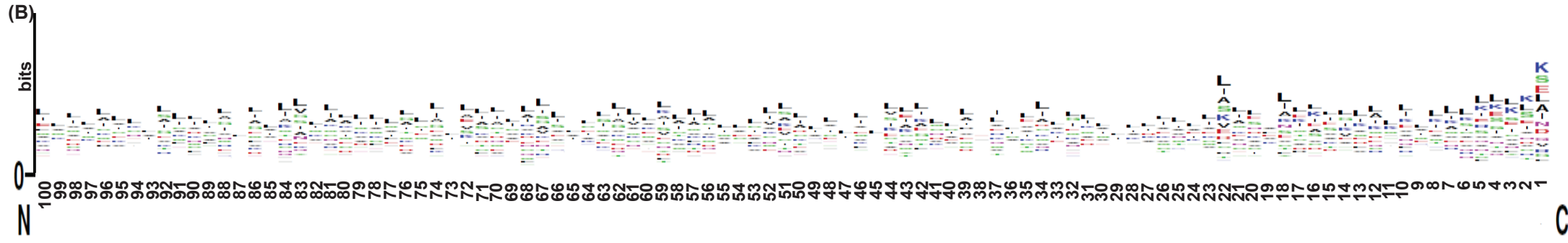

Supplement: Additional file 4: Figure S2 — Position-specific Aac profiles of T4S and control proteins for C-terminal 100 positions. The horizontal axis indicates the C-terminal position number. (A) and (B) represent T4S proteins and control proteins, respectively. [file 1471-2164-15-50-S4.pdf]

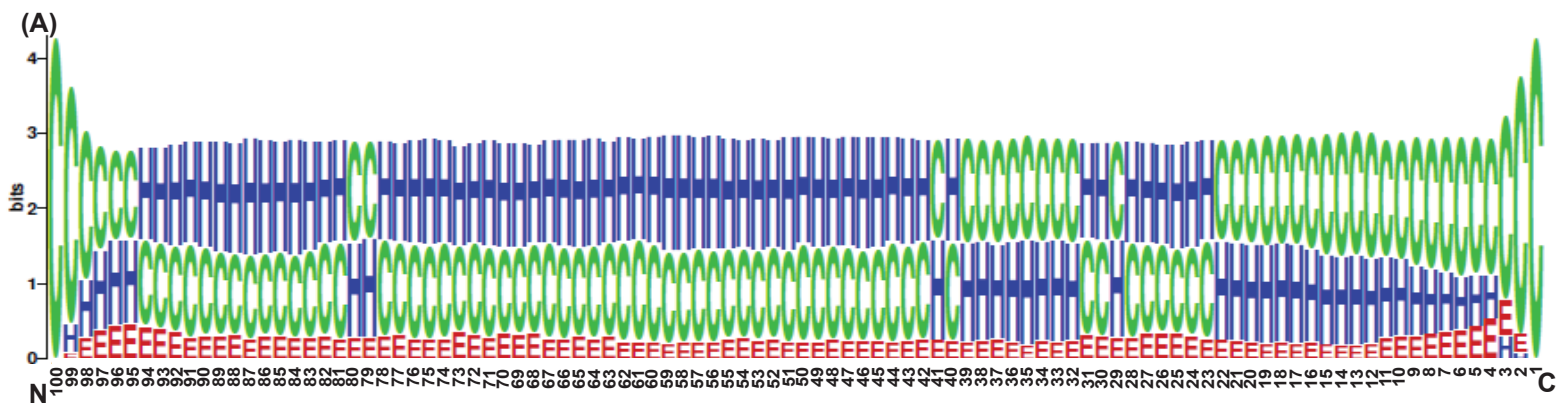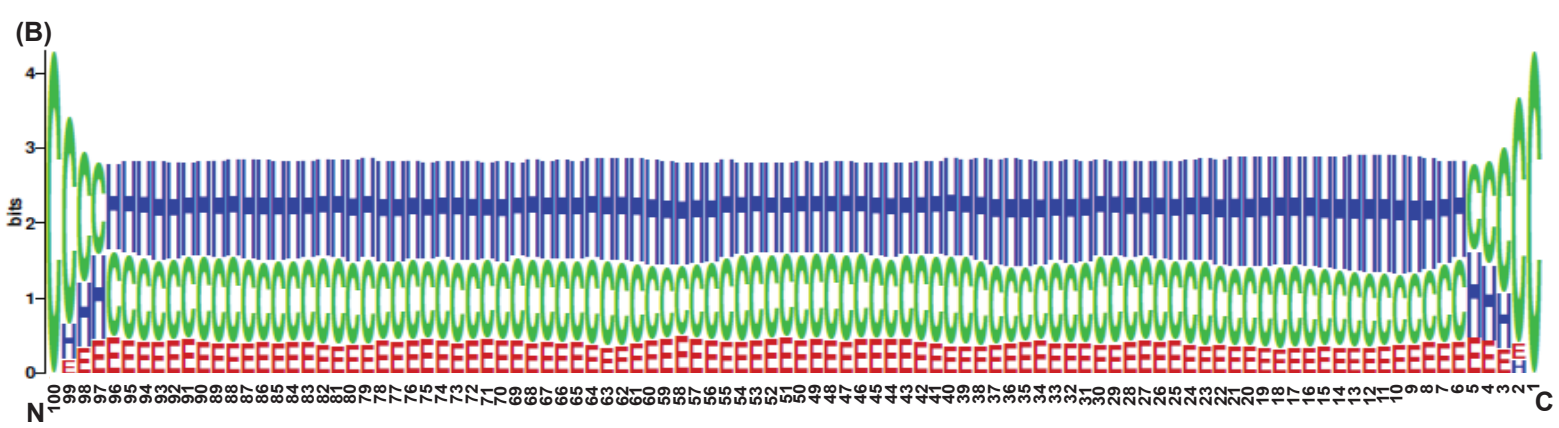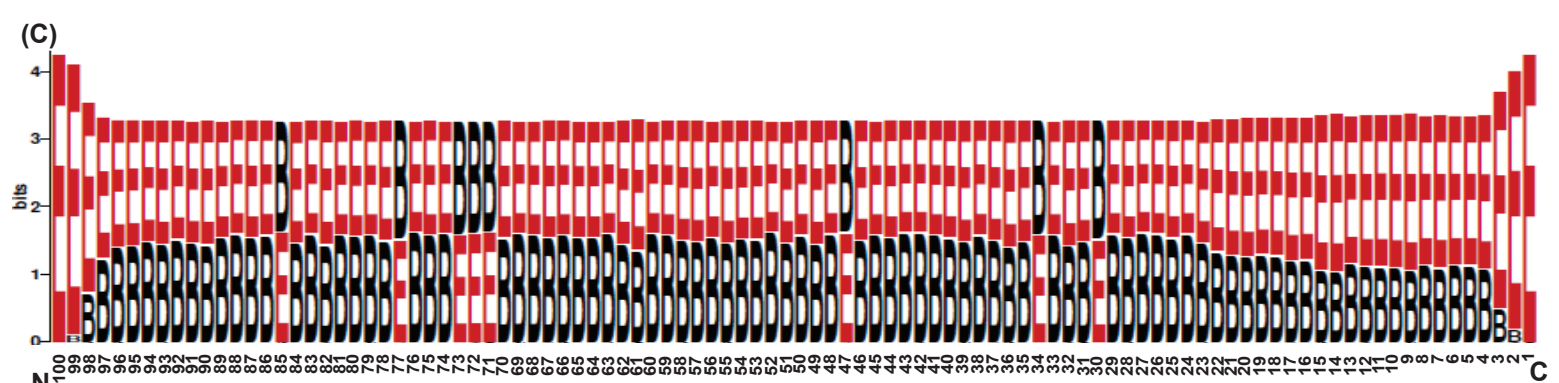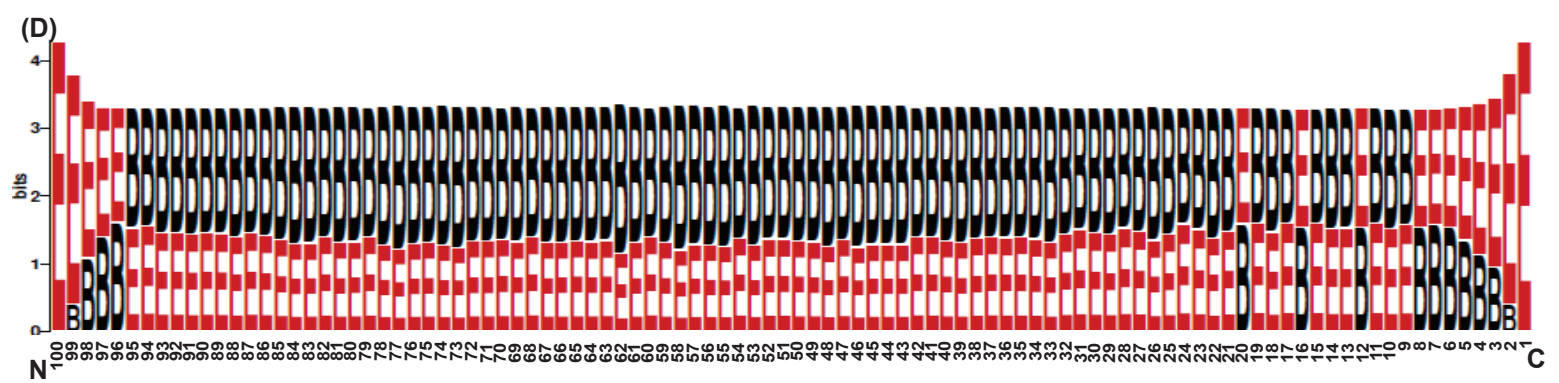

Supplement: Additional file 6: Figure S3 — Position-specific Sse and Acc profiles of T4S and control proteins for C-terminal 100 positions. The horizontal axis indicates the C-terminal position number. (A) and (B) represent the Sse of T4S proteins and control proteins, respectively. (C) and (D) represent the Acc of T4S proteins and control proteins, respectively. [file 1471-2164-15-50-S6.pdf]

(A)

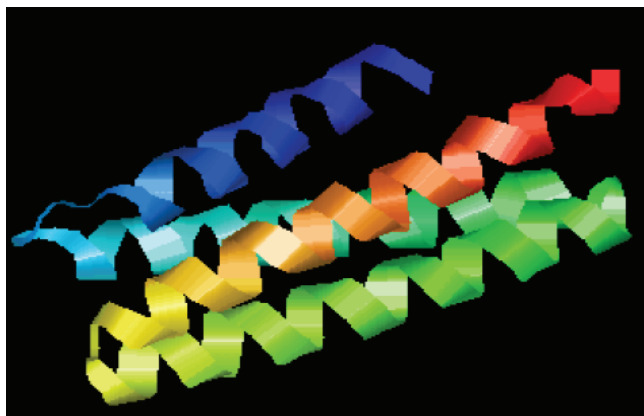

(B)

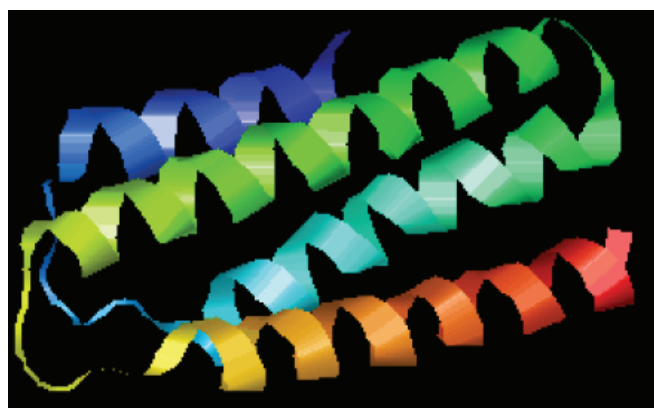

(C)

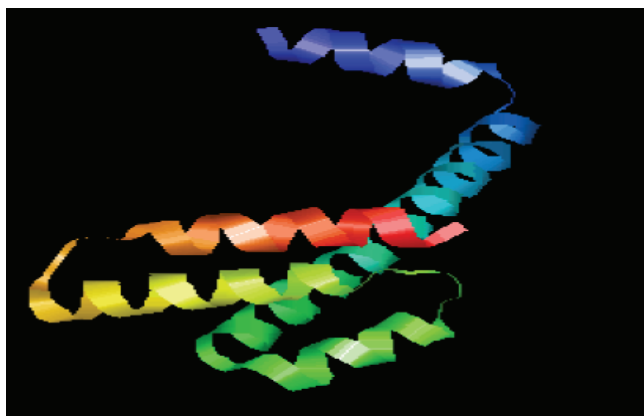

(D)

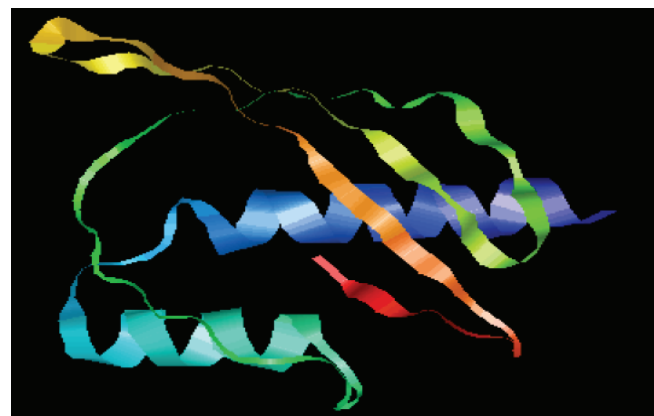

(E)

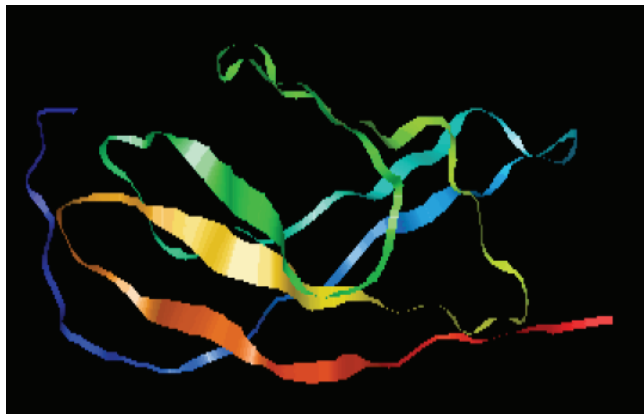

(F)

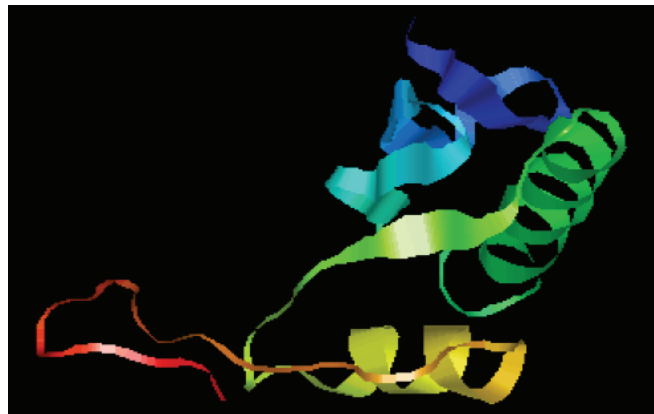

Supplement: Additional file 7: Figure S4 — 3D structure of C-terminal 100aa peptides of T4S effectors. (A)Legionella VipE; (B)Legionella YP_094180.1; (C)Legionella YP_094076.1; (D)Coxiella YP_001597263.1; (E)Legionella YP_094096.1; (F)Legionella YP_094157.1. [file 1471-2164-15-50-S7.pdf]

(A)

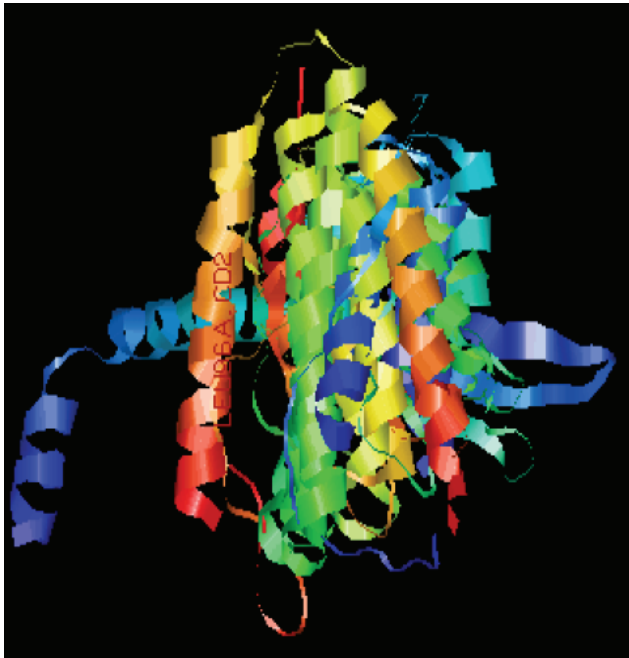

(B)

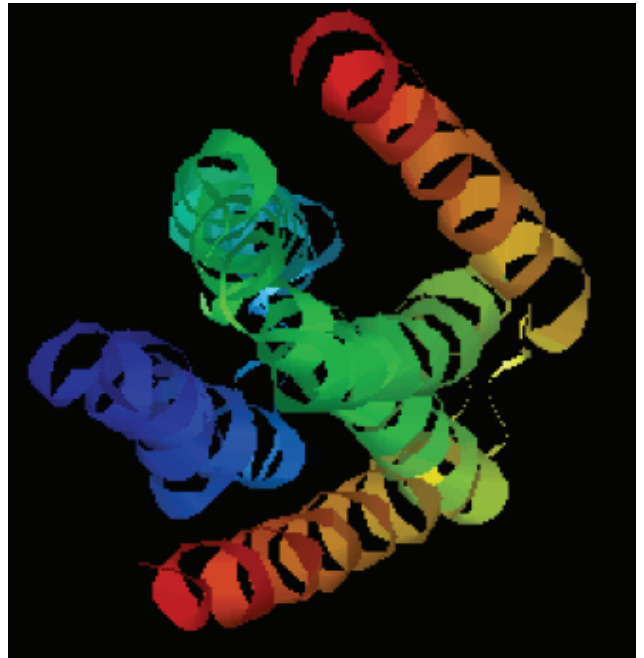

(C)

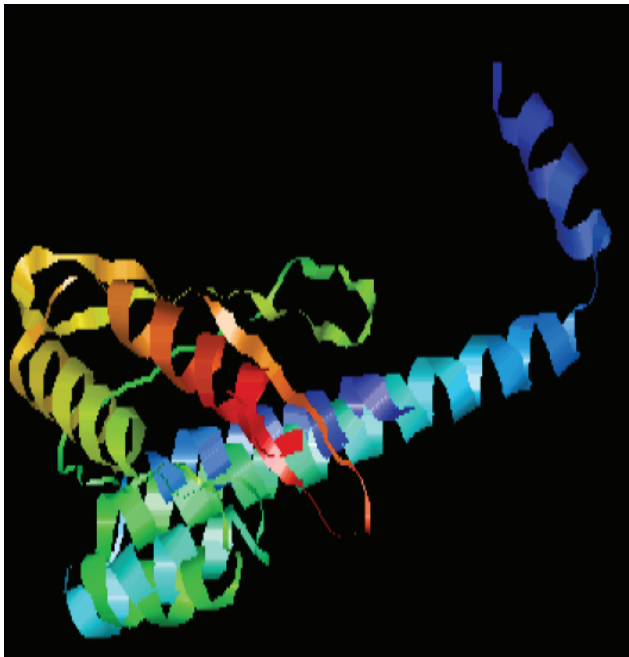

(D)

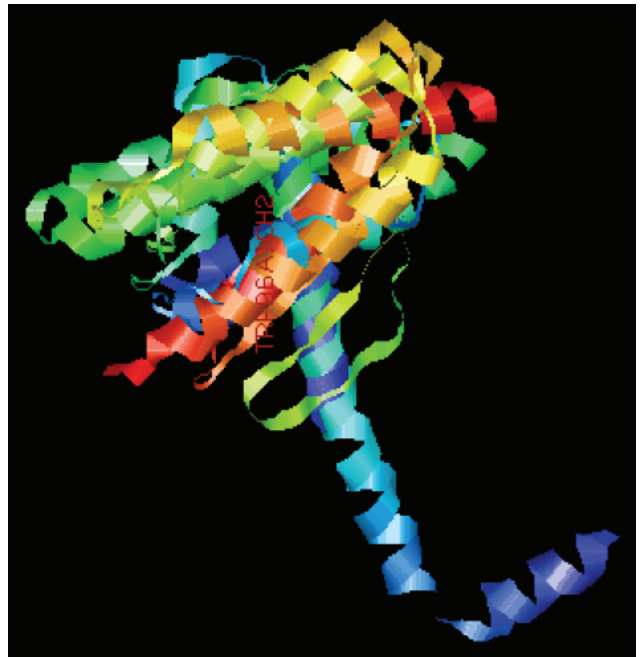

Supplement: Additional file 8: Figure S5 — Structural similarity among C-termini of T4S effectors. (A) The structure cluster formed by all the six T4S effectors with high prediction accuracy (Legionella VipE, YP_094180.1, YP_094076.1, YP_094096.1, YP_094157.1 and Coxiella YP_001597263.1); (B) Structure alignment between Legionella VipE and YP_094180.1; (C) Structure alignment between Legionella YP_094076.1 and Coxiella YP_001597263.1; (D) Structure alignment among Legionella VipE, YP_094180.1, YP_094076.1 and Coxiella YP_001597263.1. [file 1471-2164-15-50-S8.pdf]
