# Supplementary material for: Prediction of bacterial type IV secreted effectors by C-terminal features
Source: BMC Genomics. 2014 Jan 21;15:50. doi: 10.1186/1471-2164-15-50 (PMC3915618; doi:10.1186/1471-2164-15-50)
Supplement: Additional file 9: Table S4 — Optimized parameters for different SVM models classifying T4S effectors and control proteins. [file 1471-2164-15-50-S9.doc]

Tabe S4. Optimized parameters for different SVM models classifying T4S effectors and control proteins.

| **Model** | **Kernel Function** | **Cost** | **Gamma** |
| --- | --- | --- | --- |
| Seq_Aac | RBF | 2 | 0.0078125 |
| Seq_Bi_Aac | RBF | 2 | 0.00390625 |
| Seq_Aac, Bi_Aac | RBF | 1 | 0.001953125 |
| Seq_Sig | RBF | 1 | 0.015625 |
| Seq_Aac, Sse, Acc | RBF | 2 | 0.0078125 |
| Pos_Aac_SPB | RBF | 1 | 0.00390625 |
| Pos_Aac _SPB+ Seq_Aac | RBF | 8 | 0.00390625 |
| Pos_Aac_BPB | RBF | 16 | 0.001953125 |
| Pos_Aac, Sse, Acc | RBF | 16 | 0.001953125 |
