# Supplementary material for: Prediction of bacterial type IV secreted effectors by C-terminal features
Source: BMC Genomics. 2014 Jan 21;15:50. doi: 10.1186/1471-2164-15-50 (PMC3915618; doi:10.1186/1471-2164-15-50)
Supplement: Additional file 11: Table S5 — Performance of models classifying T4S effectors and non-effectors (data size ratio between negative and positive data: 6:1; 5-fold cross validation). [file 1471-2164-15-50-S11.doc]

**Table S5. Performance of models classifying T4S effectors and non-effectors (data size ratio between negative and positive data: 6:1; 5-fold cross validation)**

| **Model** | ***Sn* (%) vs. *Sp* (%)** | ***A* (%)** | ***AUC*** |
| --- | --- | --- | --- |
| T4SEpre_psAac | 79.54 vs. 94.19 | 92.10 | 0.9412 |
| T4SEpre_bpbAac | 80.98 vs. 94.33 | 92.42 | 0.9680 |
| T4SEpre_Joint | 90.78 vs. 97.65 | 96.67 | 0.9895 |

Note: The RBF kernel function was used for all three models.
