# Supplementary material for: Prediction of bacterial type IV secreted effectors by C-terminal features
Source: BMC Genomics. 2014 Jan 21;15:50. doi: 10.1186/1471-2164-15-50 (PMC3915618; doi:10.1186/1471-2164-15-50)
Supplement: Additional file 12: Table S6 — Performance of models classifying T4S effectors and non-effectors (data size ratio between negative and positive data: 6:1; 10-fold cross validation). [file 1471-2164-15-50-S12.doc]

**Table S6. Performance of models classifying T4S effectors and non-effectors (data size ratio between negative and positive data: 6:1; 10-fold cross validation)**

| **Model** | ***Sn* (%) vs. *Sp* (%)** | ***A* (%)** | ***AUC*** |
| --- | --- | --- | --- |
| T4SEpre_psAac | 78.10 vs. 94.38 | 92.05 | 0.93272 |
| T4SEpre_bpbAac | 78.96 vs. 94.67 | 92.42 | 0.9676 |
| T4SEpre_Joint | 91.07 vs. 97.74 | 96.79 | 0.9911 |

Note: The RBF kernel function was used for all three models.
